# Genetic analysis of common factors underlying cardiovascular disease-related traits

**DOI:** 10.1186/1471-2156-4-S1-S56

**Published:** 2003-12-31

**Authors:** Xiao-Qing Liu, Anthony JG Hanley, Andrew D Paterson

**Affiliations:** 1 Program in Genetics and Genomic Biology, The Hospital for Sick Children, Toronto, Ontario, Canada; 2 Division of Epidemiology and Biostatistics, Samuel Lunenfeld Research Institute, Mount Sinai Hospital, Toronto, Ontario, Canada; 3 Department of Public Health Sciences, University of Toronto, Toronto, Ontario, Canada

## Abstract

**Background:**

Cardiovascular disease-related traits, such as body mass index (BMI), systolic blood pressure (SBP), triglycerides (TG), total cholesterol (TC), high-density lipoprotein cholesterol (HDL), and glucose levels (GLUC), have moderate to high correlations with each other. We hypothesized that there might be some common factors underlying the correlations of these traits, and attempted to identify these factors and their genetic structures. Cross-sectional measurements from the 330 extended Framingham Heart Study families were used in this study. Principal component factor analysis was applied to obtain the factors that were then analyzed using variance components linkage analysis.

**Results:**

With the above six traits three factors were generated: BMI-SBP-GLUC, HDL-TG, and TC-TG. The heritabilities for these factors were 32%, 45%, and 49%, respectively. Comparing the linkage results of the factors with the results of their component traits, evidence for linkage was observed for the TC-TG factor to a locus on chromosome 2p23 with a two-point LOD score 2.73 (marker GATA8F07) and a multipoint LOD score 1.81 (at 54 cM), while the LOD scores for TC and TG did not exceed 1 at this region.

**Conclusion:**

Our analysis showed a locus on chromosome 2 might have a pleiotropic effect on the cardiovascular disease-related traits TC and TG.

## Background

High body mass index (BMI), systolic blood pressure (SBP), triglycerides (TG), total cholesterol (TC), blood glucose levels (GLUC), and low high-density lipoprotein cholesterol (HDL) are risk factors for cardiovascular disease (CVD). They are also the features of the metabolic syndrome (syndrome X) [1]. The correlations of these traits have provoked a literature documenting common factors underlying the correlations by principal component factor analysis (PCFA) – a data reduction method [2]. In order to understand the genetic structures of these factors, several groups first obtained factors from the correlated traits using PCFA, and then performed genetic analysis of these factors [3-5]. The results from these studies show that PCFA may have more power to detect the quantitative trait loci (QTLs) underlying the correlations of disease-related traits than analyses of individual traits.

In this study, we examined the relationship between the CVD-related traits, identified the underlying factors, estimated their heritabilities, and performed a genome-wide quantitative trait linkage analysis for susceptibility loci influencing the factors by using the Framingham Heart Study families.

## Methods

### Subjects and phenotypes

We used subjects from both of the Framingham cohorts. For the original cohort, most of the traits are from Exam 11 (between 1969 and 1971) while the fasting lipids are from Exams 10 to 12 (between 1967 and 1973). For the offspring cohort, the traits are from Exam 1 (between 1971 and 1975). These particular exam periods were chosen because: 1) in the Original Cohort, TG was only measured once – either at Exam 10, 11 or 12; 2) the traits from the two cohorts were collected during the same period of time. If an individual was treated for hypertension at the selected time point, his/her SBP was set to be missing. BMI was calculated as the weight in kilograms divided by the square of the height in meters. HDL, TC, TG, SBP, and BMI were log transformed, and GLUC was reciprocally transformed to minimize non-normality. Each measure was then adjusted for the effects of age, sex, age*sex, age^2^, age^3^, age^4^, number of cigarettes smoked per day, and grams of alcohol consumed per day using a multiple linear regression procedure, separately by cohort. The standardized residuals were used in the PCFA.

### Principal Component Factor Analysis

PCFA is a variable reduction procedure that extracts uncorrelated factors from correlated variables [2,6]. All analyses were performed using SAS FACTOR procedure (Version 8.2, Cary, NC) [6]. Orthogonal transformation (varimax rotation) was chosen. The common eigenvalue threshold of unity (1.0) and scree plots were used to retain the factors. The loading threshold was set to be 0.4 for factor interpretation. There were 1213 individuals from the original cohort and 1672 from the offspring cohort. However, due to missing values for some traits, there were a total of 2117 individuals (563 from the original cohort and 1554 from the offspring cohort) used for factor analysis. Coefficients of congruence were used to evaluate the similarities of factor loadings from separate analyses of the original and offspring cohorts [7].

For a valid factor analysis, the following assumptions should be met: 1) interval-level measurement; 2) random sampling; 3) linearity; and 4) normal distributions [6]. Random sampling is of the major concern for the family data we were using. In order to see if the violation of the assumption of random sampling affected our results, we repeated the factor analysis 20 times by randomly selecting one member from each family. Coefficients of congruence were calculated for the corresponding factors between the whole data and each of the random samples.

### Genetic analysis

Variance components method was applied for genetic analyses using SOLAR v 1.7.3 [8]. Heritabilities were estimated for the derived factor scores, individual traits (BMI, GLUC, SBP, HDL, TC, and TG) that comprise the factors, and other CVD-related traits (low-density lipoprotein cholesterol (LDL), the TG-to-HDL ratio (TG/HDL), the LDL-to-HDL ratio (LDL/HDL), and the TC-to-HDL ratio (TC/HDL)). LDL was calculated from the measurements of the fasting TC, HDL, and TG [9]. For all the above factors and individual traits, two-point and multipoint linkage analyses were performed with 398 autosomal markers spaced approximately at 10 cM across the genome. Ethics approval was obtained from the Hospital for Sick Children Research Ethics Board (#2002/116).

## Results

Separate correlations between the six traits (BMI, GLUC, SBP, HDL, TC, and TG) from the original cohort and the offspring cohort are shown in Table 1. Before combining the data from the original cohort and offspring cohort together, we tested the similarities of their factor loadings separately with factor analyses. Both cohorts yielded three factors accounting for approximately 68% of the total variance. The coefficients of congruence for the three corresponding factors are 0.97, 0.96, and 0.93 between these two cohorts. These indicate that the patterns of factor loadings were very similar in these two cohorts.

Table 2 provides the results of the PCFA of the combined original and offspring data. Three factors were identified. BMI, GLUC, and SBP have high loadings on factor 1, HDL and TG on factor 2, and TC and TG on factor 3. These three factors explain 69% of the original variance. The proportions of the traits' variance explained by this factor structure (communality estimates) range from 49% for GLUC to 87% for HDL.

We also investigated whether there were any marked differences between the factor loading patterns of the whole data and the random samples (by selecting one member from each family, see Methods). For the 20 random samples, the first three factors account for a mean of 69% of the total trait variance with range 66%-72%. The mean coefficients of congruence between the factors of the whole data and the factors of each of the random samples are 0.99 (range 0.98–1.00), 0.98 (range 0.94–1.00), and 0.98 (range 0.90–1.00). These results indicate that the factor structures of these random samples were very close to the structure of the whole data.

The heritabilities of the factors and individual traits are listed in Table 3. For the factors, the heritabilities range from 32% for the BMI-GLUC-SBP factor to 49% for the TC-TG factor. For the individual traits, the heritabilities range from 23% for GLUC to 50% for LDL. All the factors and individual traits are significantly heritable (*P *< 10^-7^).

The two-point linkage analysis results are summarized in Table 4. All LOD scores ≥ 2 are listed including LOD scores for other traits and factors at the same location. The strongest linkage signal for the BMI-GLUC-SBP factor is on chromosome 10, with LOD = 1.37 (marker GATA70E11). This locus is also at the same region with the strongest linkage signal for SBP (LOD = 2.06). The HDL-TG factor has two LOD scores above 2: one is on chromosome 19, which may reflect the high LOD score from HDL (marker Mfd139, LOD = 3.19); the other one is on chromosome 6q23, with its component traits HDL and TG having the LOD scores equal to 1.47 and 1.90, respectively. For the TC-TG factor, the highest LOD score (2.73) was found on chromosome 2p23.2 (marker GATA8F07), while none of its component traits have a LOD score higher than 1 at this region. This is also the most significant linkage result for any of the factors. For individual traits, the strongest evidence for linkage was observed on both chromosomes 19q13.3 (marker Mfd139) for HDL and 18p11.3 (marker GATA88A12) for TC with identical LOD scores (3.19).

**Figure 1:**
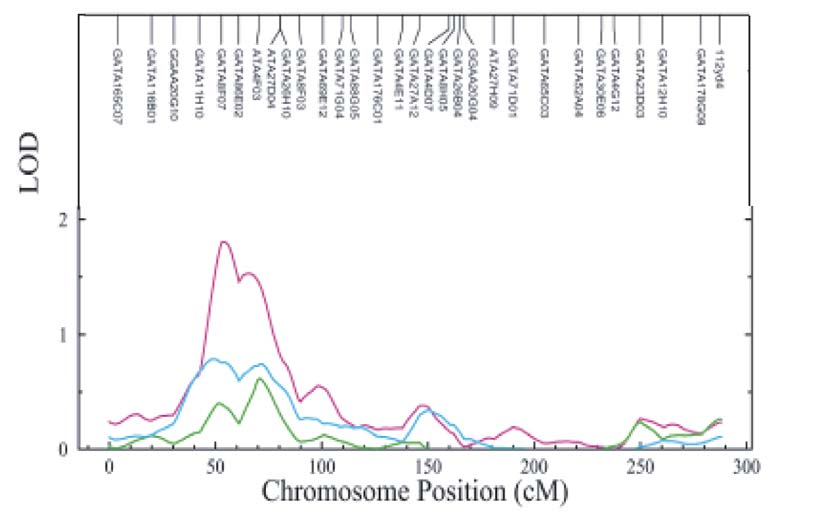
**Multipoint LOD plot for TC-TG factor, TC, and TG on chromosome 2 **Line colors: TC-TG factor (pink), TC (turquoise), and TG (green).

**Figure 2:**
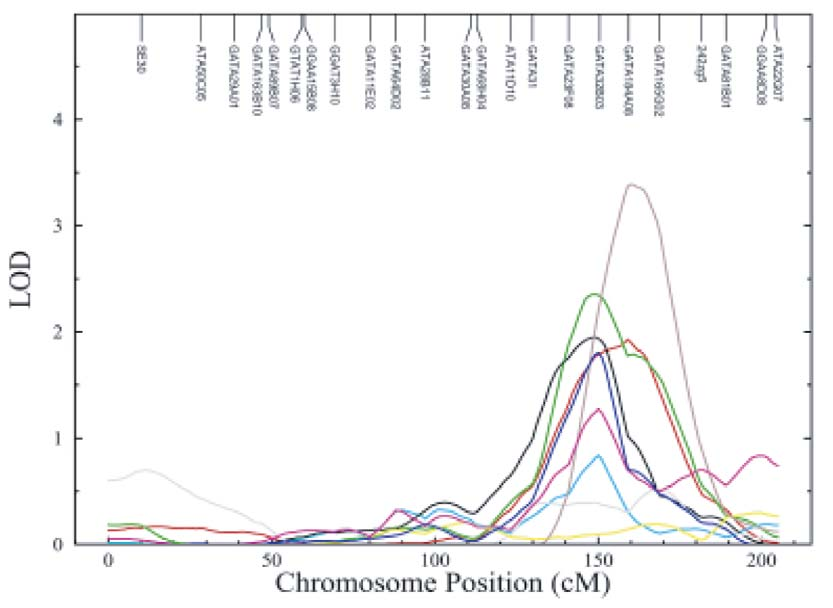
**Multipoint LOD plot for the factors and individual traits on chromosome 6 **Line colors: BMI-GLUC-SBP factor (red), HDL-TG factor (blue), TC-TG factor (pink), BMI (brown), GLUC (gray), SBP (yellow), HDL (black), TC (turquoise), and TG (green).

The results from multipoint linkage analysis are similar to the two-point linkage analysis results. In particular, Figure 1 demonstrates the multipoint results of the TC-TG factor and individual traits TC and TG on chromosome 2. The TC-TG factor peaks at 54 cM with a LOD score equal to 1.81, but the LOD scores for TC and TG are lower than 1 at this region. Figure 2 shows that the HDL-TG factor, the TC-TG factor, HDL, TC, and TG peak around 150 cM on chromosome 6, while the BMI-GLUC-SBP factor and BMI peak around 160 cM.

## Discussion

In this study, we used PCFA to investigate the clustering features of six CVD-related traits. Three factors were generated: BMI-GLUC-SBP, HDL-TG, and TC-TG. All these factors and individual CVD-related traits are under significant genetic influences. Using two-point and multipoint variance components linkage analyses, we found suggestive evidence of linkage for these factors and individual traits.

There has been another factor analysis study of the traits from the Framingham Heart Study offspring cohort [7]. In that study, the factor analysis produced one central metabolic syndrome factor (fasting and 2-hour post-challenge insulin levels, TG, HDL, BMI, and waist-hip ratio), one impaired glucose tolerance factor (fasting and 2-hour levels of glucose and insulin), and one hypertension factor (SBP, diastolic blood pressure, and BMI). Our HDL-TG factor may correspond to the central metabolic syndrome factor; the BMI-GLUC-SBP factor partially overlaps with the hypertension factor; but the TC-TG factor is a new lipid factor. The differences in the results between our study and the previous study may be due to 1) the limited number of traits that were available in this study: for example, we were not provided with the insulin measures, which are essential for defining the metabolic syndrome; 2) the handling of the covariate effects: instead of adjustment and standardization, the previous study [7] performed separate factor analyses on the subgroups stratified by age (< 60 years vs. > 60) or smoking status (current vs. nonsmokers); 3) the measurement of glucose: the glucose measurement we used was not fasting; 4) the origin of the data: 2458 subjects from Exam 5 of the Offspring Cohort were used in Meigs's study, while we used 2117 subjects from both the offspring and original cohorts.

Comparing the linkage results of the factors to the results of individual traits, most of the loci that show suggestive evidence of linkage (LOD ≥ 2) to the factors likely reflect the strong linkage signal from one of its component individual traits. The exception is the region on chromosome 2p23.2, which shows evidence of linkage to the TC-TG factor but has no high LOD score for any of the individual traits. This is also a region (35 to 58 cM) that shows the evidence of linkage to TG in the Pima Indian population [10]. This indicates that TC and TG may be influenced by a common gene (pleiotropy) on chromosome 2p. We also observed linkage signals for HDL, TC, TG, and the HDL-TG and TC-TG factors around 150 cM, and for BMI and the BMI-GLUC-SBP factor around 160 cM on chromosome 6q23. A study of nondiabetic Mexican-Americans by Duggirala et al. [11] reported that this region might contain a pleiotropic gene for TG, BMI, and insulin. For our study, since the 1-LOD support interval around the peak is very broad, it is hard to know if this region (from 130 cM to 176 cM) has one pleiotropic gene that controls the lipid traits (HDL, TC, and TG) and BMI, or whether there are separate genes for each individual trait in co-incident linkage on chromosome 6 [12].

Factor analysis has been criticized for being a subjective method due to the results being sensitive to the criteria used to determine the number of factors to choose, the factor loading threshold, and the number of variables to be included in the analysis. However, the factor loading patterns for traits related with metabolic syndrome appear to be relatively stable across demographic and metabolic subgroups [13]. For a disease like CVD, which has many intercorrelated traits, the clustering of the traits may reflect the existence of a relatively small number of factors. Identification of these factors and elucidation of their molecular basis will improve our understanding of the disease. Furthermore, some loci may have larger effects on the factors than on the individual traits, and the effect size of a locus influences the power to detect linkage.

## Conclusions

By using PCFA, we identified three independent factors underlying the intercorrelation of the CVD-related traits in a community-based study population. These factors are significantly influenced by genetic effects. Our analysis also shows suggestive evidence of linkage to chromosome 2p23.2 for a lipid factor. Since none of the results for individual traits and factors reaches genome-wide significance in this study, the improvement of the LOD score for the TC-TG factor compared to the LOD scores of its component traits may be due to chance or real pleiotropic effect. Further study is needed to explore the application of PCFA in genetic studies for closely correlated traits of common complex diseases.

## Figures and Tables

**Table 1:** Pearson product moment correlation coefficients of the traits after adjustment and standardization

	**BMI**	**GLUC**	**SBP**	**HDL**	**TC**	**TG**
BMI	-	-0.16^D^	0.24^D^	-0.22^D^	0.08^A^	0.18^D^
GLUC	-0.24^D^	-	-0.10^B^	0.16^D^	0.04	-0.17^D^
SBP	0.33^D^	-0.23^D^	-	-0.02	0.06	0.19^D^
HDL	-0.21^D^	0.08^B^	-0.03	-	0.06	-0.48^D^
TC	0.14^D^	-0.02	0.08^C^	0.10^D^	-	0.30^D^
TG	0.22^D^	-0.06^A^	0.15^D^	-0.38^D^	0.35^D^	-

Upper triangle for the original cohort, lower triangle for the offspring cohort; ^A^*P *< 0.05; ^B^*P *< 0.01; ^C^*P *< 0.001; ^D^*P *< 0.0001

**Table 2:** Factor loadings and communality of the traits

	**Factor Loadings**			
		
	**BMI-GLUC-SBP Factor**	**HDL-TG Factor**	**TC-TG Factor**	**Communality Estimates**
BMI	**0.67**^A^	-0.26	0.13	0.54
GLUC	**-0.68**	0.04	0.16	0.49
SBP	**0.74**	0.07	0.17	0.58
HDL	-0.06	**0.92**	0.16	0.87
TC	0.04	0.06	**0.92**	0.86
TG	0.11	**-0.68**	**0.56**	0.78
Eigenvalue	1.84	1.16	1.11	-
Total variance (%)	31	19	19	-
Cumulative variance (%)	31	50	69	-

^A^Factor loadings > 0.4 are in bold type

**Table 3:** Heritabilities of the factors and traits

**Trait**	**Heritability ± SE**	**Sample Size**
BMI-GLUC-SBP factor	0.32 ± 0.04	2117
HDL-TG factor	0.45 ± 0.04	2117
TC-TG factor	0.49 ± 0.05	2117
BMI	0.39 ± 0.04	2292
GLUC	0.23 ± 0.04	2474
SBP	0.24 ± 0.04	2366
HDL	0.40 ± 0.04	2380
TC	0.47 ± 0.04	2390
TG	0.42 ± 0.04	2389
LDL	0.50 ± 0.04	2360
TG/HDL	0.45 ± 0.04	2380
LDL/HDL	0.46 ± 0.04	2360
TC/HDL	0.46 ± 0.04	2380

**Table 4:** Two-point LOD scores and chromosomal positions of the factors and traits

**Chr**	**Location (cM)**	**Marker**	**BMI-GLUC-SBP Factor**	**HDL-TG Factor**	**TC-TG Factor**	**BMI**	**GLUC**	**SBP**	**HDL**	**TC**	**TG**	**LDL**	**TG/HDL**	**LDL/HDL**	**TC/HDL**
2	48	GATA8F07	0.43	0.48	**2.73**	0.64	0.01	0.39	0.15	0.72	0.75	0.19	0.49	0.05	0.06
3	119	GATA68D03	0	0.43	0.03	0	0	0	0.49	0.07	0.03	0.70	0.10	**2.50**	1.51
6	129	GATA23F08	0.56	1.13	0.28	0.02	0.05	0.08	**2.15**	0.10	1.02	0	0.89	0.23	0.54
6	138	GATA32B03	0.81	**2.09**^A^	1.28	1.40	0.09	0.12	1.47	1.19	1.90	0.46	**2.30**	0.26	0.81
6	146	GATA184A08	0.83	0.02	0.04	**2.45**	0.01	0.10	0.34	0.01	0.46	0.06	0.41	0.34	0.61
6	155	GATA165G02	0.77	0.18	0.14	**2.38**	0.43	0.36	0.02	0.02	1.07	0.15	0.69	0.01	0.07
7	155	GATA112F07	0	0.15	0.58	0	0	0	0	0.46	**2.43**	0.04	1.61	0	0
10	46	GATA70E11	1.37	0	0.07	0	0.01	**2.06**	0	0.09	0	0.04	0	0	0
18	13	GATA88A12	0	0	**2.33**	0.14	0	0.02	0.45	**3.19**	0	**2.19**	0	0.34	0.25
19	68	Mfd139	0	**2.58**	0.04	0	0	0	**3.19**	0.20	0.07	0.76	0.92	**2.83**	**3.12**
20	25	218yb5	0.10	0	**2.63**	0.77	0.17	0.31	0	1.38	0.78	0.32	0.18	0	0
20	39	GGAA7E02	0.64	0.01	0.12	0.51	0.60	0.12	0	0.24	**2.33**	0.26	1.74	0	0.09

^A^LOD scores ≥ 2 are in bold type; Chr, chromosome.
